# Giants’ cooperation: a draft genome of the giant ciliate Muniziella cunhai suggests its ecological role in the capybara’s digestive metabolism

**DOI:** 10.1099/mgen.0.001263

**Published:** 2024-07-02

**Authors:** Franciane Cedrola, Marcus Vinicius Xavier Senra, Millke Jasmine Arminini Morales, Priscila Fregulia, Lucas Canesin, Roberto Júnio Pedroso Dias, Vera Nisaka Solferini

**Affiliations:** 1 Laboratório de Diversidade Genética, Departamento de Genética, Evolução, Microbiologia e Imunologia, Instituto de Biologia, Universidade Estadual de Campinas, Campinas, São Paulo, Brazil; 2 Centro de Ciências Naturais e Humanas, Universidade Federal do ABC, Santo André, Brazil; 3 Laboratório de Protozoologia, Programa de Pós-Graduação em Biodiversidade e Conservação da Natureza, Instituto de Ciências Biológicas, Universidade Federal de Juiz de Fora, Juiz de Fora, Minas Gerais, Brazil; 4 Instiuto Tecnológico Vale, Belém, Pará, Brazil; 5 Center for Computational Engineering and Sciences, Universidade Estadual de Campinas, Campinas, São Paulo, Brazil

**Keywords:** caviomorph rodents, endosymbiotic ciliates, genomics, Trichostomatia

## Abstract

Several hundred ciliate species live in animals’ guts as a part of their microbiome. Among them, *Muniziella cunhai* (Trichostomatia, Pycnotrichidae), the largest described ciliate, is found exclusively associated with *Hydrochoerus hydrochaeris* (capybara), the largest known rodent reaching up to 90 kg. Here, we present the sequence, structural and functional annotation of this giant microeukaryote macronuclear genome and discuss its phylogenetic placement. The 85 Mb genome is highly AT rich (GC content 25.71 %) and encodes a total of 11 397 protein-coding genes, of which 2793 could have their functions predicted with automated functional assignments. Functional annotation showed that *M. cunhai* can digest recalcitrant structural carbohydrates, non-structural carbohydrates, and microbial cell walls, suggesting a role in diet metabolization and in microbial population control in the capybara’s intestine. Moreover, the phylogenetic placement of *M. cunhai* provides insights on the origins of gigantism in the subclass Trichostomatia.

**Impact Statement**

The first genome of a hindgut ciliate symbiont characterized structural and functionally, which led to insights into the ecological role of trichostomatian ciliates in hindgut fermenter hosts. Molecular phylogeny of Trichostomatia sheds light on the origins of gigantism within this subclass.

## Data Summary

The authors confirm all supporting data, code and protocols have been provided within the article or through supplementary data files.

## Introduction

Ciliates (Alveolata, Ciliophora) form a monophyletic group presenting, as a synapomorphy, two kinds of nuclei sharing the same cytoplasm: a germline and almost transcriptionally silent micronucleus and a somatic and transcriptionally active macronucleus. Micronucleus and macronucleus originate from the zygotic nucleus, with macronucleus differentiating through complex steps of large-scale DNA rearrangements and elimination, *de novo* telomere addition, and several rounds of chromosome amplification [1].

**Fig. 1.**
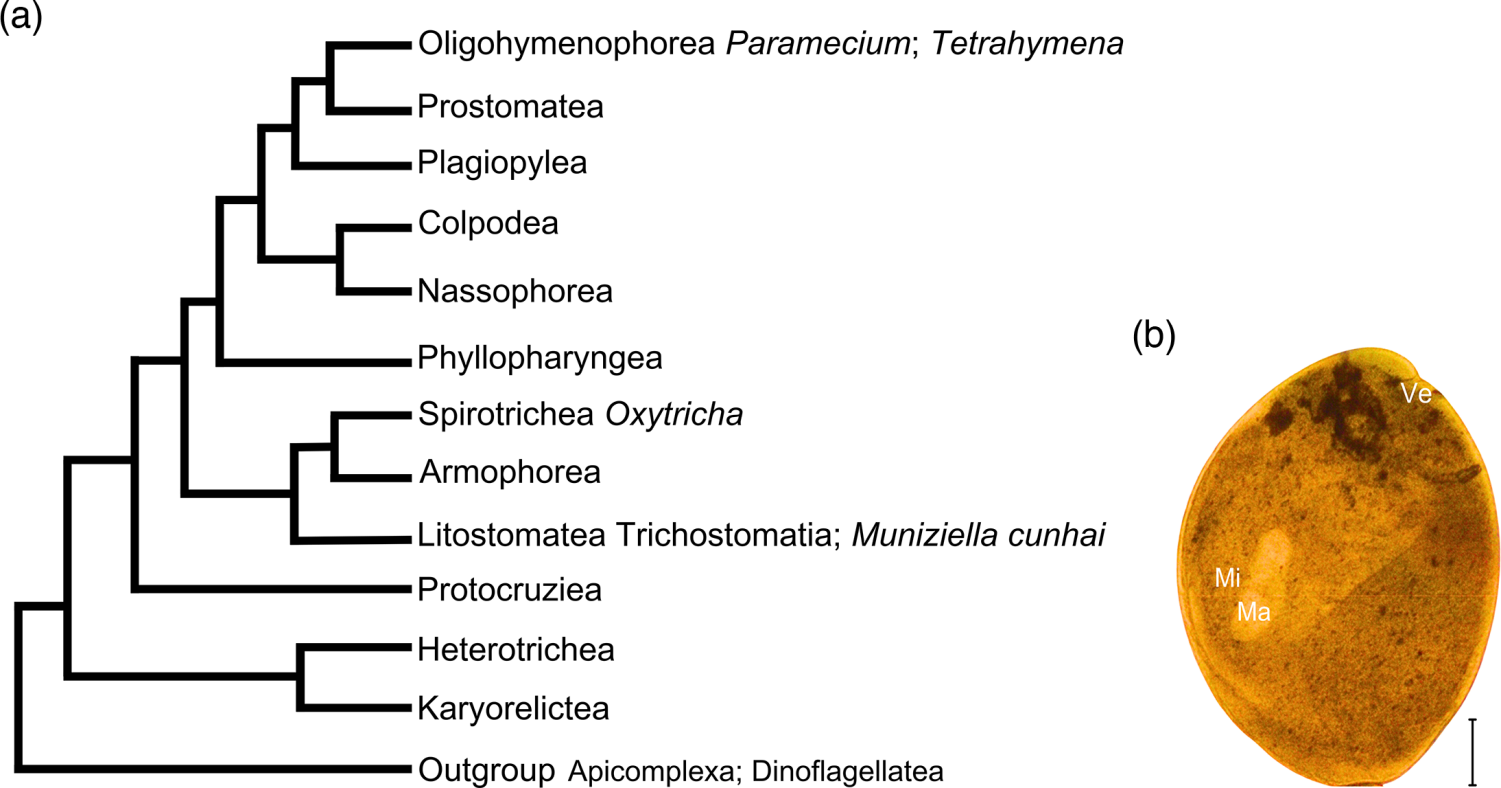
(a) Phylogenetic tree representing the class-level evolutionary relationships within the phylum Ciliophora (TSAR, Alveolata). Figure was based on [59,63]. (b). Photomicrograph of *Muniziella cunhai* Fonseca, 1939 specimen isolated in cecal content of a Brazilian capybara (*Hydrochoerus hydrochaeris* Linnaeus, 1766). Cell fixed in formalin and observed in light microscopy. Ma: Macronucleus; Mi: Micronucleus; Ve: vestibulum. Scale bar: 500 µm.

Since the first observation by Gruby and Delafond [2], several ciliate species were described as symbionts in animal gastrointestinal tracts (stomach, cecum, and large intestine). *Muniziella cunhai* (Trichostomatia, Pycnotrichidae), the largest described ciliate (around 3 mm, Fonseca [3]) (Fig. 1), is found exclusively associated with *Hydrochoerus hydrochaeris* (capybara), the largest known rodent reaching up to 90 kg [4].

*Hydrochoerus hydrochaeris* presents a fermentative chamber posterior to the small intestine, the cecum, hosting bacteria, archaea, fungi, and ciliates; such a complex microbiome is considered adapted to a diet of grass, aquatic plants, fruits, and tree barks [4,5]. As observed in other mammals, capybaras regularly ingest a special type of faeces (cecotrophes) produced in the cecum, a behaviour known as cecotrophy. The cecotrophes are rich in short-chain fatty acids (SCFAs), microbial protein, vitamins, and minerals and are supposed to provide additional nutrients that were not fully absorbed during the initial digestion of their food [4].

Ciliates from capybara’s cecum belong to two orders of the class Litostomatea, subclass Trichostomatia: the order Entodiniomorphida, mainly represented by species of the family Cycloposthiidae, and the order Vestibuliferida, represented by ciliates from the families Hydrochoerellidae, Protohallidae, and Pycnotrichidae (Fig. 1) [6,9].

The family Pycnotrichidae was proposed by Poche [10] to include the species *Pycnothrix monocistoides*, mainly based on the presence of a full body-length vestibulum. Subsequently, Corliss [11] expanded this family to include the genera *Buxtonella; Collinina; Infundibulorium; Muniziella* and *Taliaferria*. Lynn [12] further added the genus *Vestibulongum* and recognized *Buxtonella* as *incertae sedis*. Despite the classification of Pynotrichidae genera being based on the presence of a long vestibulum, they exhibit a significant phenotypic diversity [12,13]. In fact, as pointed by Cedrola *et al*. [14], the long vestibulum appears to be a homoplastic character within the subclass Trichostomatia. Interestingly, among the ciliates of the family Pycnotrichidae, only *Muniziella cunhai* and *Pycnothrix monocystoides* exhibit large body sizes (gigantism) and consequently share common morphological features, such as the thick ectoplasm composed of numerous layers. To date, molecular data for pycnotrichiid ciliates are only available for *Pycnothrix monocystoides* [13] and *Infundibulorum cameli* [15]. However, the monophyly of the family Pycnotrichidae has not yet been assessed through molecular phylogenetic reconstructions.

Microorganisms inhabiting the gastrointestinal tracts of herbivorous mammals exhibit various adaptations to the gut environment. These adaptations include an anaerobic lifestyle and the capacity to metabolize carbohydrates and release compounds that can be absorbed by the host [16]. Concerning ciliates, numerous microscopic and molecular studies have provided insights into these adaptations, notably the presence of Mitochondrial Related Organelles (MROs). These organelles share similarities to mitochondria but may have distinct functions or characteristics [17]. Although we can observe microorganisms in symbiosis with several mammalian hosts, most studies are on rumen representatives and these microbes are poorly understood in hindgut fermenters. In a recent genomic study of capybara’s cecal microbiome, Cabral *et al*. [5] presented the role of Eubacteria in breaking down structural carbohydrates. Although ciliates constitute most of this microbiome biomass [8], their functional role remains unknown; so far, studies have focused mainly on their morphology and systematics [7,9,18].

Our aims were to elucidate the phylogenetic placement of *M. cunhai*, to explore the origins of gigantism in the subclass Trichostomatia and, based on the functional genomic features of *M. cunhai*, we make inferences on its role on the digestive metabolism of capybaras and the nature of their symbiotic relationship.

## Methods

### Sample collection, morphological characterization, DNA extraction, amplification, and sequencing

*Muniziella cunhai* was obtained from the cecal contents of a single host (*H. hydrochaeris*) hit by a car; the specimen was made available by the Instituto Brasileiro do Meio Ambiente e dos Recursos Naturais Renováveis (IBAMA), in Juiz de Fora, Minas Gerais, Brazil. Ciliates were isolated under a stereomicroscope using glass micropipettes and immediately fixed in absolute ethanol. Also, a small sample was preserved in 18.5 % formalin for morphological characterization and taxonomic confirmation. Only empty individuals, with clear internal contents, were selected for DNA extraction. For details, see Cedrola *et al*. [8].

Pools of 30 ciliates were used for total DNA extraction using the DNeasy Blood and Tissue kit (QIAGEN), following the animal tissue protocol. Genomic DNA was amplified using the REPLI-g Single Cell kit (QIAGEN) and the product was sent to CD Genomics Service Company (https://www.cd-genomics.com/). The macronuclear genome was sequenced in a hybrid approach, based on short reads (2×150 bp, Illumina MiSeq) and on long reads (average 10 kb, maximum 100 kb, MinION, Oxford Nanopore).

### Genome assembly, complete and partial chromosome identification, and genetic code inference

After inspection using FastQC v.011.9 (http://www.bioinformatics.babraham.ac.uk/projects/fastqc/), a quality control protocol was performed with fastp [19], using default parameters. Only reads with a Q score >20 for every base were selected for subsequent analyses. Cleaned short and long reads were used to assemble the genome, using SPAdes v. 3.15.5 with the following parameters [-t -m --sc -k 21,33,55 --pe1-1 --pe1-2 --nanopore -o] using a hybrid assembling strategy [20]. Assembling statistics were obtained using QUAST v. 5.0.2 [21] with default parameters, and BUSCO v. 4.0.6 [22] against the Alveolata OrthoDB v. 10 [23] was employed to estimate overall genome completeness.

To exclude potential contamination, only scaffolds/contigs carrying the typical telomeric sequences of Litostomatea ciliates in at least one of their ends were considered for functional annotation [24,26]: the ends (50 bp) of each scaffold were screened for telomeric repeats using the software MEME v. 4.12.0 [27]. Scaffolds capped with at least 1.5 times the telomeric sequence at both ends (complete chromosomes) and at only one end (incomplete chromosomes) were filtered for the subsequent analyses, using a python script modified from [24].

The genetic code and codon usage were inferred with FACIL [28] and with the Sequence Manipulation Suite web server (https://www.bioinformatics.org/sms2/codon_usage.html), respectively, both using default parameters.

### Gene prediction and annotation

Non-coding RNAs were annotated with Infernal v. 1.1.4 [29] and Aragorn [30], with default parameters. Genes were predicted using BRAKER2 applying the protein data mode [31], suitable for genomes without RNA-Seq evidence data, using the OrthoDB Protozoa v. 10 [23] as protein evidence.

Functional annotation was performed with EggNOG-mapper v. 2.0 [32,33] using the Diamond mapping mode [33] and against three functional databases: Cluster of Orthologous Genes (COG), Gene Ontology (GO), and Kyoto Encyclopaedia of Genes and Genomes (KEGG). Carbohydrate-Active enzymes (CAZymes) were annotated using the dbCAN3 meta server [34] against the CAZy database v. 9.0, which is a dedicated database for genomic, structural, and biochemical analyses on CAZymes.

### Phylogenetic analyses

#### Phylogenomic

A phylogenomic analysis was performed using all (18) litostomatean genomes/transcriptomes available in the GenBank database (May 2023) (in group: subclass Trichostomatia [*n*=17]; outgroup: *Didinium nasutum*) (File S1, available in the online version of this article), using Orthofinder [35]. Orthofinder assigned 207 891 genes (76.4 % of total) to 41 347 orthogroups. Fifty percent of all genes were in orthogroups with four or more genes (G50 was four) and were contained in the largest 12 045 orthogroups (O50 was 12045). A gene tree was inferred based on each orthogroup using -M msa -T fasttree parameter; a rooted species tree was estimated from all inferred gene trees using STAG and STRIDE methods [36,37], both implemented in Orthofinder.

### 18S rDNA sequences and tree topological test

The 18S rDNA sequences of 133 litostomatean species were retrieved from the GenBank database (May 2023). This set of 182 sequences (in-group: Trichostomatia [*n*=181]; outgroup *Spathidium papiliferum*) (File S1), were joined to the *M. cunhai* sequence obtained from the Infernal analysis (see Gene Prediction and Annotation section for details). Alignments were constructed using the sina software [38], with default parameters. After manual inspection, regions of ambiguous nature and primer sequences were trimmed, producing an equi-length data set (1721 bp). The best model of sequence evolution was inferred using the JModel-Test v. 2.1.4 software [39,40]. Bayesian inference (BI) analysis was performed with MrBayes v. 3.2 [41] with the evolutionary model GTR +G+I. Two simultaneous and independent Markov Chain Monte Carlo simulations were performed using three heated chains and one cold chain (N chains=4) with a sample and print frequency of 500 and a diagnostic frequency of 5000, for 1 000 000 generations or until the average standard deviation of the split frequencies was below 0.01, indicating convergence of the two independent runs. All remaining trees after discarding the burn-in (25 %) were used for calculating the posterior probabilities, using a majority rule consensus. Maximum likelihood (ML) analysis was carried out by using the RAxML software v. 8.2.4 [42], applying a GTR +G+I nucleotide substitution model. The reliability of internal branches was assessed using the rapid bootstrap method with 1000 replicates.

KH (Kishino–Hasegawa) tree topological test was conducted in IQTree software [43], using the likelihood-based δ statistic to compare the best ML topology (unconstrained tree), obtained in our phylogenetic analysis with an alternative phylogenetic hypothesis that assumed the monophyly of the family Pycnotrichidae (constrained tree).

**Fig. 2.**
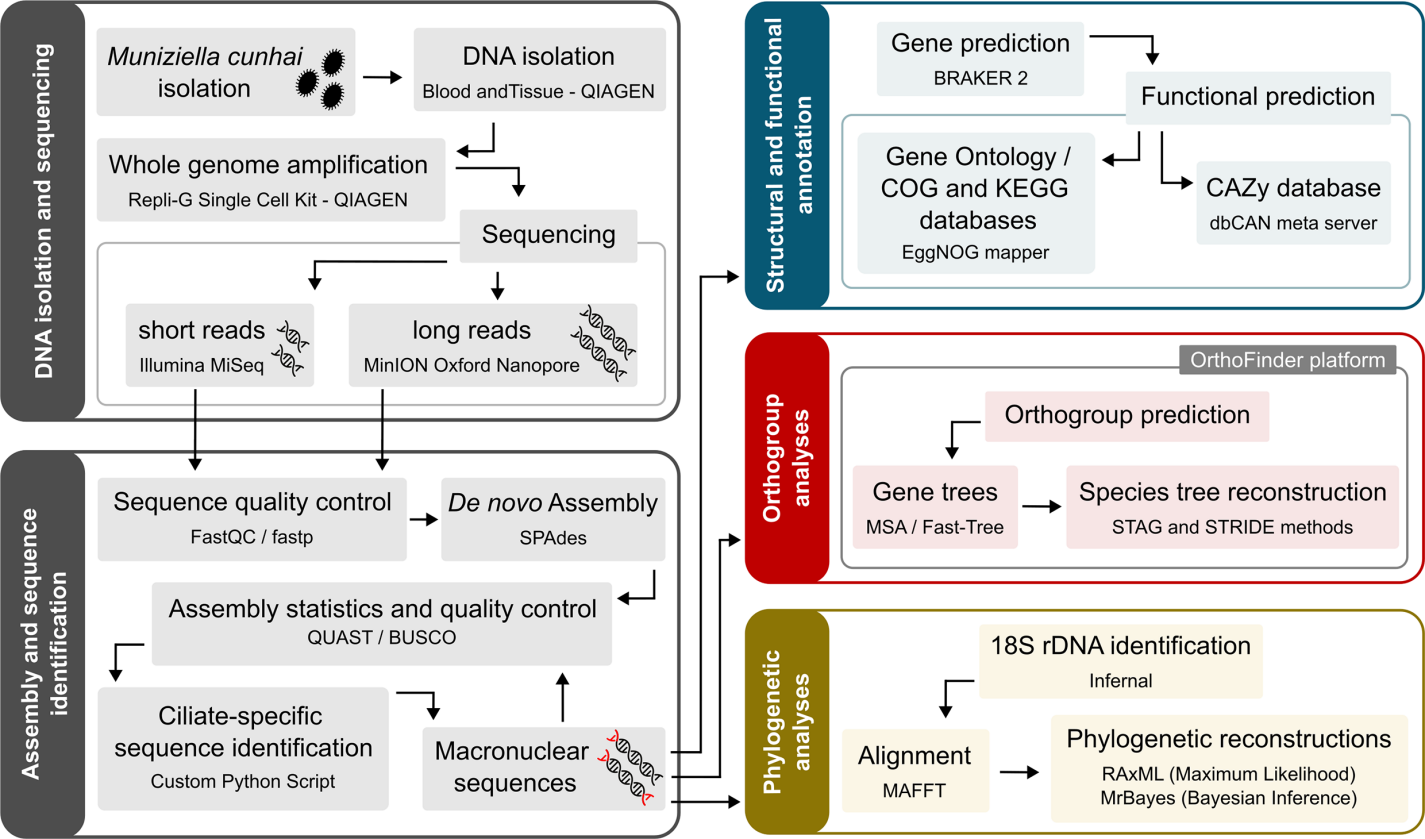
Pipeline for genome assembly, gene prediction and annotation; phylogenomic and phylogenetic reconstructions developed in this study.

The overall experimental and analyses procedures are summarized in Fig. 2.

## Results

### Morphological features of *Muniziella cunhai* (Fig. 1 and File S2)

*Muniziella cunhai* was described by Fonseca [3], primarily based on live observations of a Brazilian sample. Batisse [44] re-examined Fonseca’s material and provided a new description of the species using histological sections stained with ferric hematoxylin, eosin, and light green. In addition to other Brazilian reports [8], the species had been documented in samples from Venezuela [45] and Bolivia [6].

In our samples we could observe all the main diagnostic characters of *M. cunhai*: body completely covered by dense short cilia (~5 µm in length); broad longitudinal groove filled with cilia (the vestibulum), extending from the anterior region to the last third of the body; very dark and granular cytoplasm containing contractile and food vacuoles, as well as food particles such as plant fibres and prokaryotes; a rod-shaped macronucleus, around 600 µm in length, consistently located in the anterior third of the body; micronucleus in a depression on the surface of the macronucleus.

### Phylogenetic analyses

**Fig. 3.**
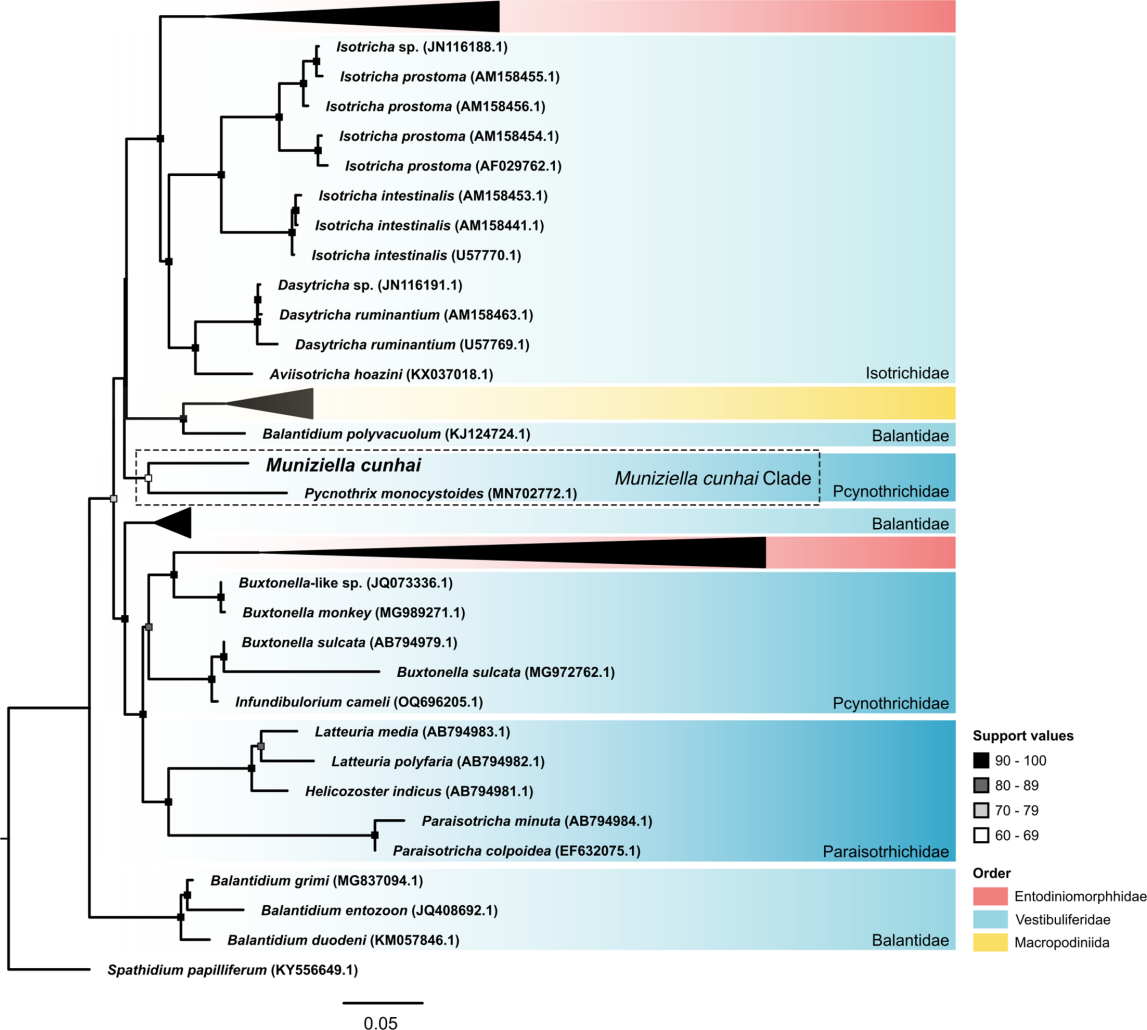
Phylogenetic tree of the subclass Trichostomatia (Ciliophora, Litostomatea) based on 18S rDNA sequences. *Spathidium papiliferum* was chosen as the outgroup. The scale bar corresponds to five substitutions per 100 nucleotide positions.

The phylogenetic trees are presented in Fig. 3 (18S rDNA) and File S3 (phylogenomic). In the phylogenomic tree, *M. cunhai* emerged as a sister taxon of all other trichostomatids while in the 18S rDNA tree it emerged as a sister taxon of *Pycnothrix monocystoides*.

### Assembly and structural annotation of *M. cunhai*

**Fig. 4.**
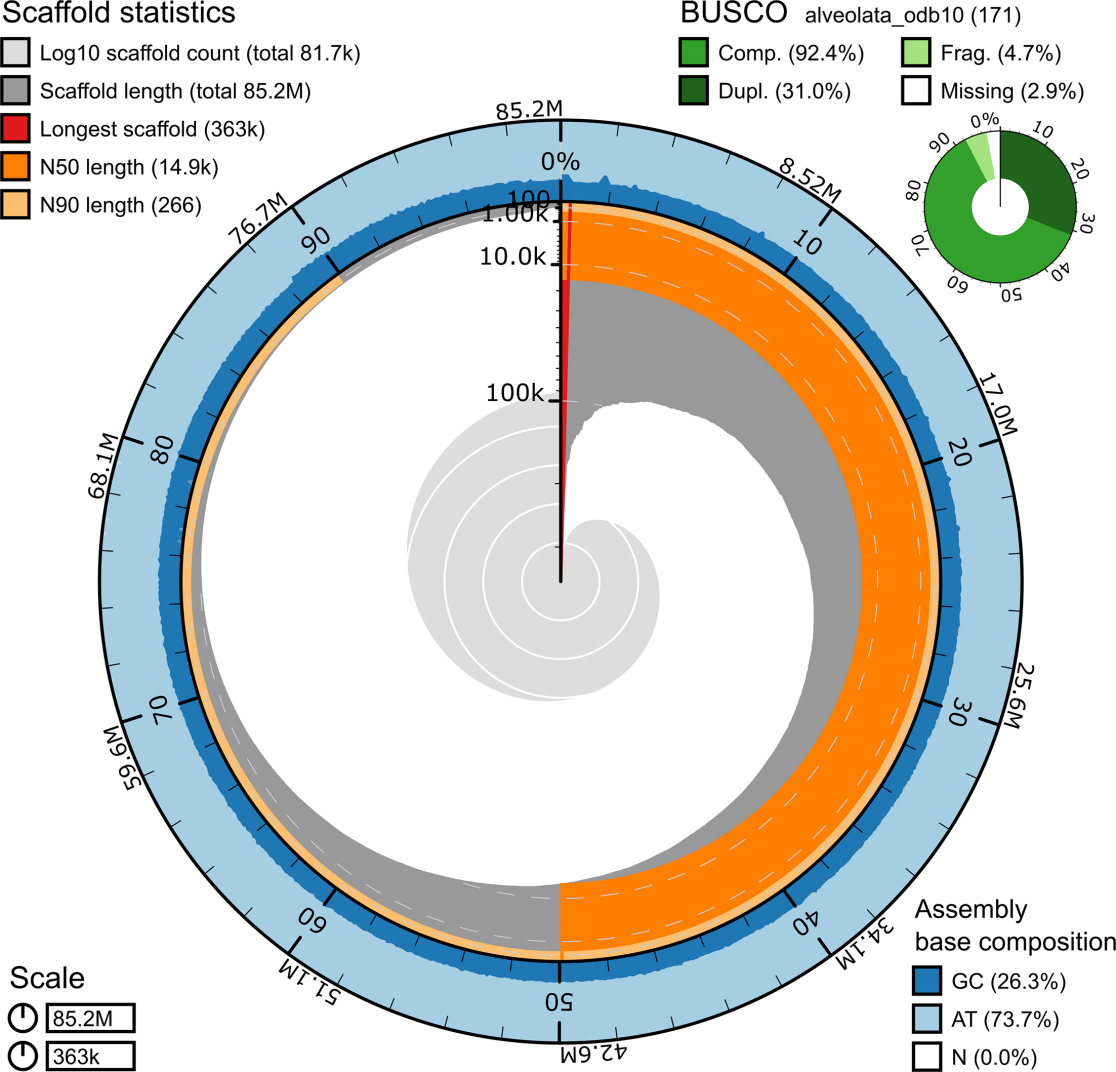
Snail plot describing the assembly statistics of the genome of *Muniziella cunhai*. From inside to outside: the central light grey spiral shows log scaled scaffold count with white scale lines marking changes order of magnitude; dark grey segments represent scaffold length distribution with plot radius scaled to the longest scaffold (red line); the dark orange segment represents N50 scaffold length; the light orange segment represents N90 scaffold length; outer blue and light blue rings show GC and AT percentages along the genome. Benchmarking Universal Single-Copy Orthologs for Alveolata database is in the upper right corner. Comp.: complete BUSCOs; Dupl.: duplicated BUSCOs; Frag.: fragmented BUSCOs; Missing: missing BUSCOs. The plots were generated using https://github.com/rjchallis/assembly-stats.

The genome sequencing provided 9 806 706 short (150 paired-end) Illumina reads and 334 448 long (~10 Kb) MinION reads. After trimming and quality control, 9 316 371 and 195 592 short and long reads, respectively, were used in a hybrid approach to assemble the genome. The draft genome assembly resulted in 15 336 scaffolds (N50=14.9 kbp, and L50=79 kbp; mean coverage of 60.7), indicating a genome length of ~85 Mb with an overall assembly completeness of 92.4 % and a relatively low GC content, estimated in 26.3 %. The chromosomes of this species are capped with telomeric sequences containing the motif (CCCCAAT)n. Among the assembled scaffolds, 1917 presented the telomeric repeats at one (1537) or both (380) ends (hereafter named as incomplete and complete chromosomes, respectively). The mean length of the complete ones is ~28 631 bp, including ~6 genes with introns with ~100 bp and an intron/gene ratio of 0.62. The genome features of *M. cunhai* are presented in Fig. 4.

Genome assembly and all associated raw data was deposited in GenBank under BioSample SAMN41686637.

**Fig. 5.**
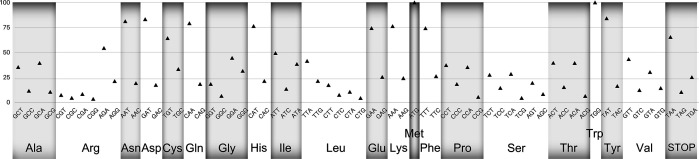
Relative frequencies of each of the 64 codons used in the macronuclear genome of *Muniziella cunhai*.

*Muniziella cunhai* presented the Standard Genetic Code without stop codons reassignments. However, a significant bias toward AT rich codons (> 70 %) was detected and TAA stop codon is the most used (> 50 %) (Fig. 5). We found 36 tRNAs corresponding to 20 standard amino acids.

### Functional annotation

**Fig. 6.**
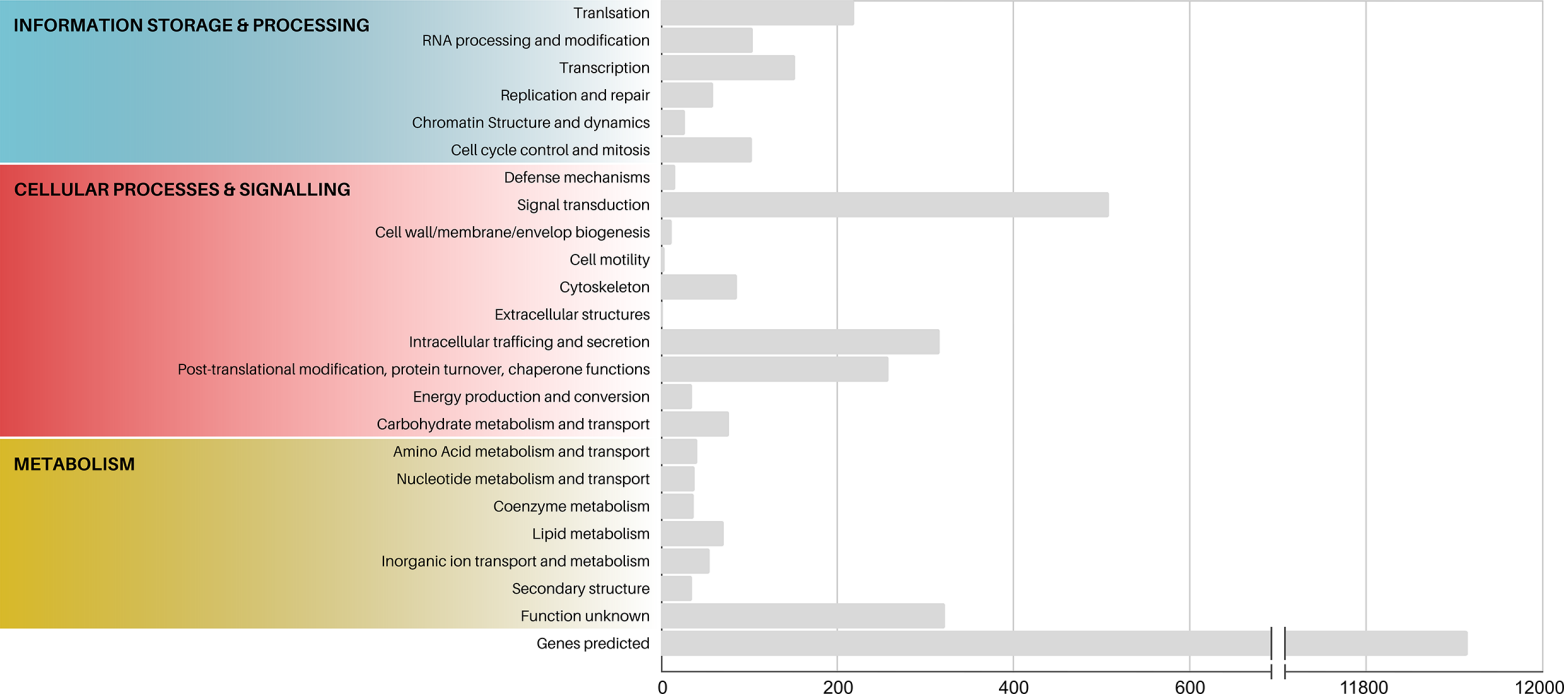
Cluster of Orthologous Genes (COGs) categories annotated in the macronuclear genome of *Muniziella cunhai*.

Based on the 11 397 predicted genes after analysis using BRAKER2, the genome of *M. cunhai* was functionally annotated using EggNOG, which classified the proteins according to different (COG, GO, and KEGG) databases. Data on EggNOG functional annotations are summarized in the File S1 and in Fig. 6.

COG database (Fig. 6; File S1) – 2600 of the predicted genes were assigned to one of 23 categories. Category T – Signal Transduction – was the most representative in the macronuclear genome, followed by categories S – Function Unknown; U – Intracellular Trafficking and Secretion and O – Post-translational Modification, Protein Turnover, and Chaperone Functions (Fig. 6).

GO database (File S1) – 2654 of the predicted genes were assigned to 101 non-redundant terms, organized into the three GO domains: the Biological Processes domain was the most representative, comprising on average 49.37 % of the total terms, followed by the Cellular Component (10.32 %) and Molecular Function (6.57 %).

KEGG database (File S1) – 2654 genes were assigned to 767 functional orthologs (KOs) and mapped to 337 KEGG Pathways involved in Metabolism, Genetic Information Processing, Environmental Information Processing, Cellular Processes, Organismal Systems, Human Diseases, and Drug Development.

We highlighted that several of these genes are related to digestive metabolism, suggesting an important role of *M. cunhai* capybara’s digestive processes.

**Fig. 7.**
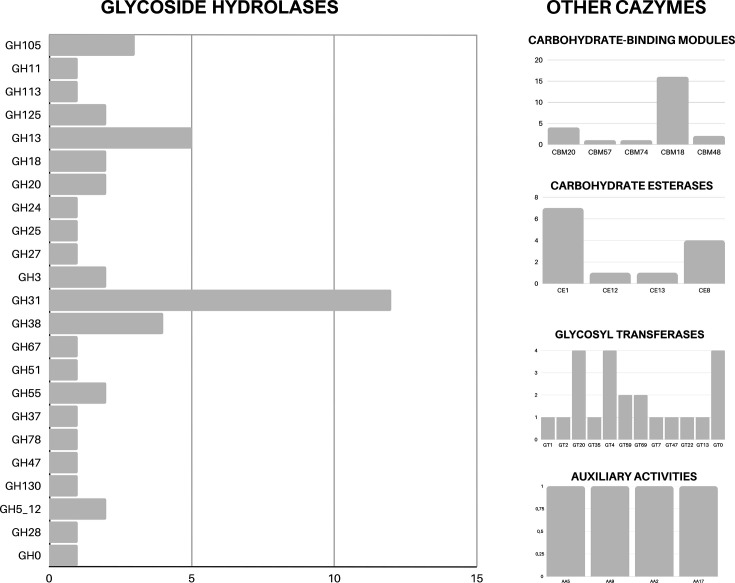
Carbohydrate active enzymes (CAZymes) annotated in the macronuclear genome of *Muniziella cunhai*.

**Fig. 8.**
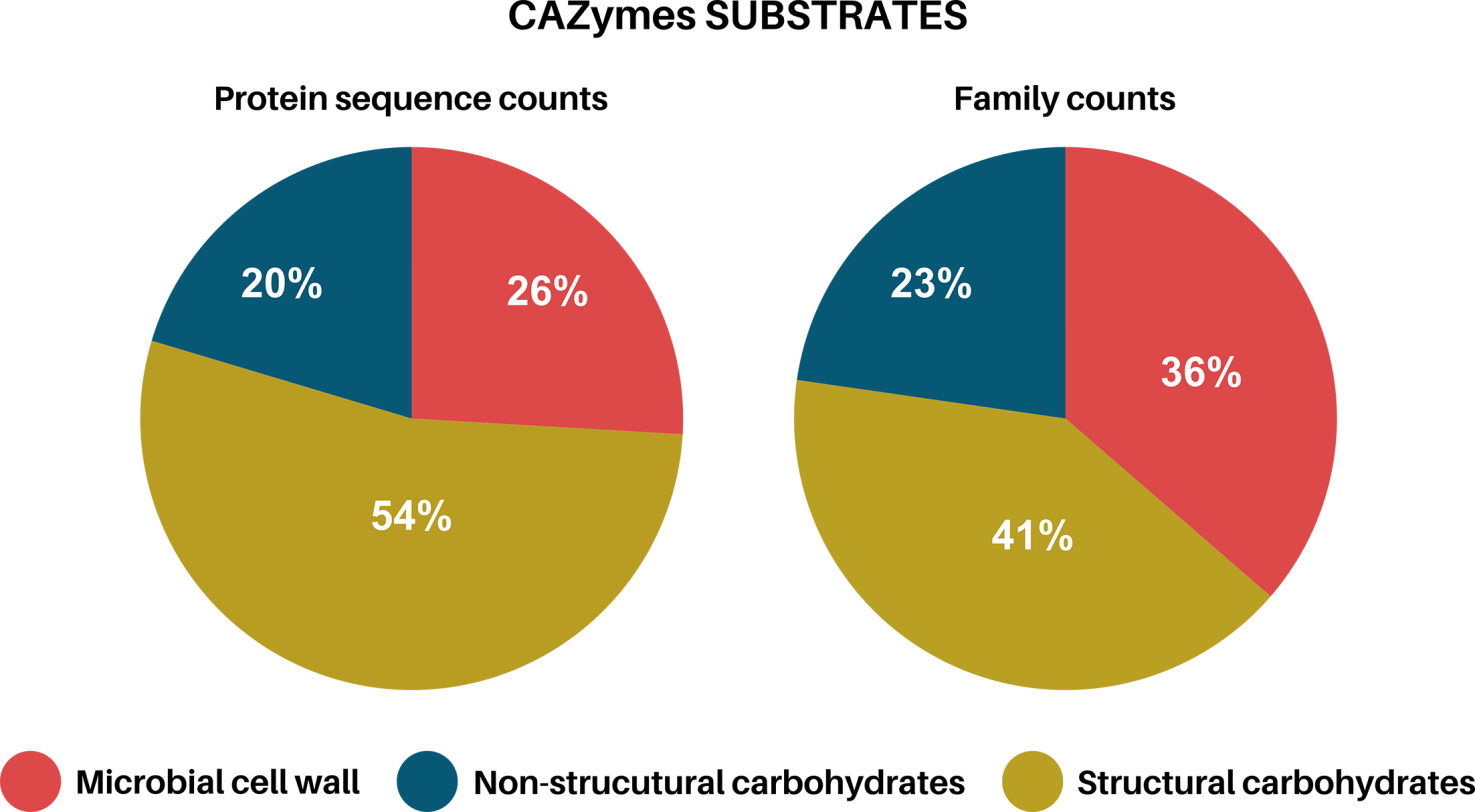
Relative proportion of the food substrates used by *Muniziella cunhai*.

We also annotated the predicted genes with dbCAN3 meta server, against CAZy database to investigate the ability of *M. cunhai* to metabolize carbohydrates. A total of 113 CAZymes (Fig. 7; File S1), attributed to 43 families of Glycoside Hydrolases (GHs), Carbohydrate-Binding Modules (CBMs), Carbohydrate Esterases (CEs), Glycosyl Transferases (GTs), and Auxiliary Activities (AAs), involved in carbohydrates breakdown and syntheses were identified (Fig. 8; File S1). The most representative were GHs, mainly those involved in cellulose and hemicellulose breakdown (Figs7  8) and those specialized in degradation of microbial cell wall carbohydrates (chitin and peptidoglycan) and starch (Figs7  8); the annotated CAZymes of CBMs and CEs families presented similar functions.

Considering the CAZymes specialized in synthesis (GTs), the substrate most utilized is starch, which is commonly utilized in glycogen synthesis.

In the annotated genome, five new CAZymes were detected, attributed to unknow families (GT0 and GH0) and in the family GT69, based on their properties (File S1).

Several of these enzymes were accompanied by signal peptides (File S1).

## Discussion

### Phylogenetic analyses and the origins of gigantism in Trichostomatia

In the phylogenomic reconstruction with data from 18 genome/transcriptome sequences, *M. cunhai* emerged as sister taxon of all other trichostomatids, consistent with peculiar morphological features observed in this species.

In the 18S rDNA phylogeny, that comprises 182 sequences, *M. cunhai* emerged as a sister taxon of *Pycnothrix monocystoides*. Based on morphological taxonomy, both species currently belong to the family Pycnotrichidae together with other five species. However, according to our phylogenetic 18S rDNA phylogeny (Fig. 3) and complementary KH tree topology test (File S1), this family is not monophyletic, suggesting that its putative synapomorphies might represent homoplastic or plesiomorphic characters in the subclass Trichostomatia [2,11, 12, 41].

The phylogenetic relatedness of *M. cunhai* and *P. monocystoides* along with their big sizes, allows us to hypothesize on the origins of their gigantism. *Pycnothrix monocystoides* and *M. cunhai* are symbiotic organisms associated with different mammals exhibiting distinct geographical distributions. *P. monocystoides* is commonly found in association with *Procavia*, a genus of the order Hyracoidea, distributed across Africa and western Arabia [13,46]. On the other hand, *M. cunhai* is exclusively isolated from Capybara rodents (Caviomorpha, Rodentia) in South America [46]. Belonging to the infraorder Hystrichognathi, caviomorphs have origins traced back to Asia or Africa, as indicated by paleontological data. They are believed to have migrated to South America via Africa or Antarctica during the Middle or Late Eocene [47]. Considering these distribution patterns, it is plausible to suggest that the last common ancestor of *Munziella* and *Pycnothrix*, along with the emergence of gigantism in Trichostomatia, may have originated in Africa or Asia.

### Genome organization of *M. cunhai*

The size of the draft macronuclear genome of *M. cunhai*, (~85 Mb) is in the range observed in other species of Trichostomatia (~30–100 Mb) [25,26].

The draft macronuclear genome is organized in chromosomes of approximately 28 000 bp, considered ‘large-sized’, a characteristic shared with the genus *Isotricha*, (chromosomes~13 360 bp long, Li *et al*. [26]). Other trichostomatians in the genus *Dasytricha* and in the family Ophryoscolecidae present chromosomes approximately 1500 bp in length, considered ‘nano-sized’ or ‘gene-sized’ [25,26]. So far, there are no hypotheses on the origins of distinctive genomic architectures in the phylum Ciliophora. However, since such variation has been reported within different classes, it seems to be homoplastic and to have evolved independently [48,49].

### Ecological role of *M. cunhai* in capybara digestive metabolism

The symbiotic association of ciliates and mammals is considered a very important evolutionary step, associated with an efficient use of dietary constituents [50]. Despite this, most of what is known about the metabolic capabilities of these ciliates come from studies on rumen symbionts, mainly from common and cultured Ophryoscolecidae species [25,26, 51, 52]. Data on hindgut symbionts are basically absent from the literature.

The high density of *M. cunhai* in the cecal content of cabybaras (~5×10^4^ cells ml^−1^) [8] led us to investigate the details of this symbiotic relationship by mining information from genomic data. *Muniziella cunhai* represents the first trichostomatid hindgut fermenter symbiont characterized in a genomic perspective and provides insights into the symbiotic relationship of trichostomatids with their hosts.

The genes involved in several signalling pathways, might respond to both internal and external stimuli. Several belong to well-known eukaryotic pathways controlling main cellular processes: transcription, translation, ribosome biogenesis, cell growth, proliferation and differentiation, cytoskeletal organization and dynamism, chemotaxis, metabolism, secretion, calcium homeostasis, cell fate, apoptosis, cell-cycle control, and oxidative stress resistance. According to Firkins *et al*. [52], in rumen ciliates these pathways might be related to rapid responses to different diets and may directly impact the host digestive metabolism. The rumen is a complex environment, with a diverse microbiota composed of bacteria, archaea, fungi, and ciliates in constant interaction with each other and with the hosts’ gastrointestinal tract, with effects on their metabolism and physiology. There is no information about these processes in hindguts, but considering that the cecum microbiota resembles the rumen’s, *M. cunhai* signalling pathways are probably essential to its performance.

In the same way, genes related to post-translational modification, protein turnover, and chaperone functions, may be related to rapid responses to different diets. Since capybaras’ foraging options are seasonally variable [4], a plastic microbiota would provide rapid adjustments to shifting diets, being nutritionally adaptive.

One of the most important roles played by the gastrointestinal microbiome of herbivorous mammals is the carbohydrate digestion. These microorganisms can metabolize both structural (plant and microbial cell walls) and non-structural carbohydrates, releasing large amounts of nutritive compounds that can be absorbed by the host [53]. Despite this, most of the knowledge about this capacity is restricted to the rumen environment [52], with few data on the hindgut microbiota [5].

In our analyses, we identified several Carbohydrate-Active Enzymes (CAZymes) specialized in the breakdown of non-structural, structural, and microbial cell wall carbohydrates. The presence of these enzymes suggests that these carbohydrates are important for *M. cunhai* energy metabolism and may also benefit the host. Hindgut ciliates belong to the same subclass (Trichostomatia) as the rumen ones [52], for which there are plenty of information; so, it is reasonable to assume that both rumen and hindgut ciliates have similar metabolic pathways. Rumen ciliates convert complex carbohydrates – mainly starch, cellulose, hemicellulose, chitin, and peptidoglycan – into simpler hexoses (e.g. glucose), that are metabolized into pyruvate and/or phosphoenolpyruvate through glycolytic pathways. Pyruvate and phosphoenolpyruvate lead to the production of short-chain fatty acids (SCFAs), such as acetate, lactate, propionate, and butyrate, that might be released in gastrointestinal tract of the host [25,52, 54]. These pathways were confirmed in our annotations: besides CAZymes, we observed several genes belonging to the Pyruvate Metabolic Pathway (e.g. K00025; K00873; K13979).

Even though SCFAs are metabolic by-products of ciliate activity, they play a pivotal role in host metabolism. In capybaras, hindgut SCFAs are available to the host in the cecotrophe and represents a primary energy source for these animals. Considering the typically low nutritional quality of herbivorous diets, the presence of large amount of SCFAs in cecotrophe is very important. Moreover, recent research has highlighted the role of SCFAs in anti-inflammatory and anti-tumorigenic actions in mammals [55].

In addition to participating in ciliate and host energetic metabolism, enzymes specialized in microbial cell wall carbohydrates degradation suggest that *M. cunhai* is a predator of bacteria and fungi in cecal environment. Besides CAZymes, Andersen *et al*. [56] identified several KEGG Orthology (KO) related to microbial cell predation in proteome of *Entodinium caudatum*. Some of these KOs were observed in our functional annotations (e.g. K03282; K18584; K18464; K18466; K18468; K07897; K02145; K02146; K02149; K12383). The predatory activity, in addition to contributing to the control of microbial populations in the cecum, enriches the cecotrophes with microbial proteins, which represents a significant part of the absorbed amino acids by the host during the cecotrophy behaviour [4]. Rodents that perform cecotrophy, including capybaras, employ a ‘mucus-trap’ mechanism to separate fine particles (including microbes) from larger indigestible material in their hindgut. In this process, microbial protein is captured in a colonic groove and moved from the proximal colon into the cecum. The material retained in the cecum is excreted separately as ‘soft faeces’ or ‘cecotrophs’, ingested directly from the anus preventing the waste of valuable microbial nutrients that would otherwise be excreted [56,58].

The genomic analysis of *Muniziella cunhai* sheds light on its role in the capybara’s digestive processes, suggesting a contribution to diet metabolization and microbial population control within the animal’s intestine, in a mutualistic relationship with the host. The close phylogenetic relationship with *Pycnothrix monocystoides* supports traditional taxonomic studies and provide valuable insights on the origin of the gigantism in the subclass Trichostomatia.

## supplementary material

10.1099/mgen.0.001263 Uncited Fig. S1.

10.1099/mgen.0.001263 Uncited Table S1.

10.1099/mgen.0.001263 Uncited video 1.
