# Supplementary material for: Giants’ cooperation: a draft genome of the giant ciliate Muniziella cunhai suggests its ecological role in the capybara’s digestive metabolism
Source: Microb Genom. 2024 Jul 2;10(7):001263. doi: 10.1099/mgen.0.001263 (PMC11316547; doi:10.1099/mgen.0.001263)
Supplement: Uncited Fig. S1. [file mgen-10-01263-s001.pdf]

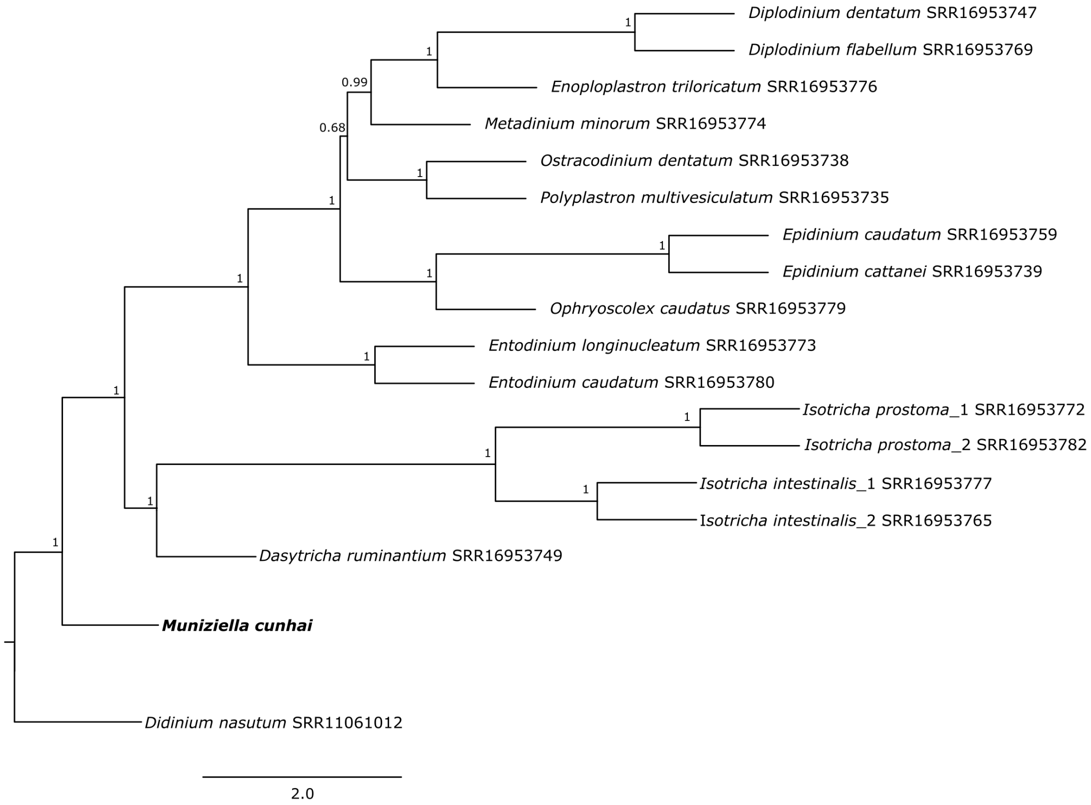

Fig. S1. Phylogenomic tree of the subclass Trichostomatia (Ciliophora, Litostomatea). *Didinium nasutum* (Haptoria) was chosen as the outgroup. The values on the branches represent the maximum likelihood (ML) bootstrap values.
